# Vaping patterns, nicotine dependence and reasons for vaping among American Indian dual users of cigarettes and electronic cigarettes

**DOI:** 10.1186/s12889-019-7523-5

**Published:** 2019-09-02

**Authors:** Dorothy A. Rhoades, Ashley L. Comiford, Justin D. Dvorak, Kai Ding, Michelle Hopkins, Paul Spicer, Theodore L. Wagener, Mark P. Doescher

**Affiliations:** 1 Department of Medicine, University of Oklahoma Health Sciences Center, and Stephenson Cancer Center, 655 Research Parkway, Room 449, Oklahoma City, OK 73104 USA; 2 Epidemiology, Cherokee Nation, Tahlequah, OK USA; 3 College of Public Health, University of Oklahoma Health Sciences Center, Oklahoma City, OK USA; 4 Center for Applied Social Research, University of Oklahoma, Norman, OK USA; 5 Oklahoma Tobacco Research Center, Stephenson Cancer Center, University of Oklahoma Health Sciences Center, Oklahoma City, OK USA; 6 Department of Family Medicine, University of Oklahoma Health Sciences Center, and Stephenson Cancer Center, Oklahoma City, OK USA

**Keywords:** Electronic cigarettes, Smoking, American Indian, Adult, Tobacco use

## Abstract

**Background:**

The American Cancer Society discourages the dual use of electronic cigarettes (ECs) and cigarettes because such use has not resulted in reduced exposures to the harmful effects of smoking. American Indian (AI) people have the highest prevalence of smoking and of EC use in the United States, but very little is known about dual EC and cigarette use in AI communities.

**Methods:**

In 2016, 375 adult AI in Oklahoma who smoked cigarettes completed a survey about EC use (vaping). We describe vaping patterns, nicotine dependence, and reasons for EC use among the subset of 44 (12%) current dual EC users. To differentiate habitual EC users from occasional or merely curious users, we defined dual use as using ECs on some days or every day in the past 30 days.

**Results:**

About one-third of dual users vaped ten or more times daily. About two-thirds used a tank product. Eleven percent used ECs without nicotine and another 9% were unsure of the nicotine content. A minority (40%) enjoyed vaping more than smoking, and most (76%) would smoke first on days they did both. Thirty-one percent vaped within 5 min of waking and another 24% within 30 min. Although the two-item heaviness of use index did not differ significantly between smoking and vaping, the ten-item Penn State Dependence Index (PSDI) suggested greater dependence on smoking than vaping (11.02 vs. 6.42, respectively; *p* < .0001). The most common reasons for vaping were to reduce smoking (79%), enjoyment of flavors (78%), and ability to vape where smoking is not allowed (73%). Perceptions of less harm to others (69%) or to self were the next most common (65%). Fewer than half used ECs to reduce stress, for affordability, or because others used them.

**Conclusions:**

Nearly 20% of dual users used ECs either without nicotine or without knowing if the product contained nicotine. The PSDI indicated greater dependence on smoking than vaping. Reasons for vaping were nearly equal between smoking reduction and enjoying flavors. Understanding patterns of dual use will inform future efforts to address nicotine dependence for AI communities with high prevalence of smoking.

## Introduction

The prevalence of smoking in the U.S. is decreasing [1] and the prevalence of electronic cigarette (EC) use increasing [2]. Many individuals use ECs, or vape, in effort to reduce or quit smoking [3–5]. While complete substitution of ECs for cigarettes has been associated with smoking cessation [6, 7], concomitant or “dual” use of ECs and cigarettes has not [8], and dual use is now the most common form of EC use [9]. Further, dual use has not been shown to reduce exposure to the harmful products of combustible cigarettes, including carcinogens [10–18]. The American Cancer Society, as a result, strongly discourages the dual use of EC and cigarettes [19]. Additionally, recent evidence suggests that frequent vaping itself carries increased risk of cardiovascular disease [20, 21], and newer generation EC devices can achieve nicotine levels comparable or exceeding those of conventional cigarettes [22], increasing the potential for adverse effects from nicotine.

The American Indian (AI) population in general has a very high smoking prevalence, particularly in the Plains states [23–26]. AI people also have the highest prevalence of EC use of any single-race group in the U.S. [27, 28]. However, use of EC by AI who smoke has been reported only rarely [29, 30], and the patterns and preferences of EC use among AI dual users, never. We now describe patterns, EC dependence, and preferences by focusing on the dual user subset, defined as people who smoked cigarettes and used ECs on some days or every day of the past 30 days. This common definition [31] minimizes the inclusion of people who merely experimented with ECs once or twice, and allows focus on persons with more habitual EC use.

## Methods

The “Vaping among Smokers: A Cherokee Nation Cohort Study” was designed to provide estimates of the prevalence and patterns of EC use among adult AI smokers and has been described in detail elsewhere [30]. Briefly, in 2016 we recruited 375 adult AI men and women, who smoke, at a large Cherokee Nation Health Services outpatient facility in northeastern Oklahoma to participate in the study. Eligibility for services includes proof of AI or Alaska Native (AN) descent, such as a Certificate of Degree of Indian Blood (CDIB), from a federally recognized AI or Alaska Native tribe or community. Eligibility to participate in the cohort included being age 18 years or older, smoking at least 100 cigarettes in one’s lifetime, smoking in the past 30 days, and answering “yes” to both “Are you American Indian?” and “Do you have a CDIB card?”

Participants completed a survey including patterns of smoking and EC use. The current cross-sectional descriptive analysis is limited to the subset of dual users as defined below.

### Measures

All participants reported whether they ever used ECs, and if so, whether they used any in the past 30 days [30]. Among these, dual users were defined as using EC on some days or every day within the past 30 days. This definition of dual use reduces the chance of including persons whose vaping was limited to curiosity or brief experimentation [31].

### Vaping measures

Dual users reported how many times per day they used an EC (categorized as: less than 5, 5–9, 10–14, 15–19, 20–29, or 30 or more), which type of product most often used (cigalike, tank, mods, other), whether their e-liquid contained nicotine (yes, no, don’t know/not sure) and the nicotine content of their usual EC product (0 mg, 1–12 mg, 13–17 mg, 18+ mg, don’t know/not sure). Dual users also reported whether they enjoyed vaping more than smoking, and whether they use EC vs a cigarette first on days they use both.

Vaping dependence and smoking dependence were separately assessed using the Heaviness of Vaping Index [32], Heaviness of Smoking Index [33] and the Penn State Dependence Index (PSDI) for vaping or smoking [32] as adapted slightly for this study (Appendix). The Heaviness of Vaping and Heaviness of Smoking indices are two-item measures of nicotine dependence for EC users and smokers, respectively. The PSDI for vaping and PSDI for smoking are 10-item indices to measure dependence on vaping or smoking, respectively. Cases with any missing component of the index were excluded from the scoring to reduce bias towards low dependence [32].

Participants indicated one or more reasons for using ECs, including to reduce cigarette smoking, liking the flavors, using when smoking is not allowed, less harmful to self than smoking, less harmful to others than smoking, reducing stress, better affordability, and/or other people important to them use ECs, or other (write in).

### Analysis

Categorical data are represented by count (percent). Continuous data are represented using the mean (SD). Dependence scales were assessed for normality using the Shapiro-Wilk test and visual confirmation via quantile-quantile plots, then compared between vaping and smoking using paired t-tests with complete case analysis. The Shapiro-Wilk test is a commonly-applied method to assess normality assumptions underlying parametric statistical procedures [34, 35]. All analyses were performed using SAS software v9.4 and R v3.5.1.

## Results

Of the 375 enrolled participants, 44 (12%) were dual users, defined as using ECs on some or all of the past 30 days.

Table 1 shows that among dual users, about one-third vaped 10 or more times a day, and less than one-half vaped fewer than 5 times per day. “Cigalikes” were the least frequently used product and tank systems were the most frequently used product. While 80% indicated that their e-liquid contained nicotine, 11% used e-liquid without nicotine, and 9% were unsure or did not know if their e-liquid contained nicotine. The most frequently reported nicotine content was 12 mg or less, but 14% did not know the nicotine concentration. More than half did not find vaping more enjoyable than smoking and most reported smoking before vaping on days they did both.

**Table 1:** Vaping patterns and preferences, American Indian EC dual users (*N* = 44)

	Frequency
Frequency of EC use per day	
Less than 5 times per day	46%
5–9 times per day	20%
10 or more times per day	34%
Type of EC product used most often	
Cigalike	12%
Tank	68%
Mods	18%
Other	2%
E-liquid contains nicotine	
Yes	80%
No	11%
Don’t know/Not sure	9%
Usual E-liquid nicotine content	
0 mg	7%
1–12 mg	39%
13–17 mg	18%
18 or more mg	23%
Don’t know/Not sure	14%
Enjoy vaping more than smoking	
Yes	40%
No	60%
On vaping days, which is used first	
Cigarette	76%
EC	24%

*EC* Electronic Cigarette

Vaping and smoking dependence item measures and scales are shown in Table 2. Although 76% reported smoking before vaping as noted above, vaping or smoking within the first 5 min of waking was not infrequent for either habit (31% and 24%, respectively), and cumulatively, the frequency of vaping or smoking within 30 min of waking was the same (55%). Individual measures show more dependence on smoking than vaping. Heaviness of vaping index and heaviness of smoking index did not differ significantly among the dual users (*p* = 0.22), but the mean PSDI dependence score for vaping was significantly lower than that for smoking (6.4 +/− 4.8 vs 11.0 +/− 5.0, respectively; *p* < .0001). Results were unchanged when median values of the PSDI were compared via the Wilcoxon signed-rank test (6 vs 11, *p* < .0001).

**Table 2:** Vaping dependence and smoking dependence measures among adult AI dual EC and cigarette users (*N* = 44)

Vaping Dependence Measures		%	Smoking Dependence Measures			%
Minutes after waking use first EC			Minutes after waking use first cigarette			
5 or less		31%	5 or less			24%
6–30		24%	6–30			31%
31–120		24%	31–120			38%
121+		21%	121+			7%
Nights per week wake to use EC			Nights per week wake to smoke			
Never/less than weekly		73%	Never/less than weekly			54%
1 to 2		12%	1 to 2			23%
3 or more		15%	3 or more			23%
Continue using EC because hard to quit			Continue to smoke because hard to quit			
Yes		44%	Yes			80%
No		56%	No			20%
Ever have strong cravings to use EC			Ever have strong cravings to smoke			
Yes		33%	Yes			84%
No		67%	No			16%
How strong were urges to vape, past week			How strong were urges to smoke in past week			
None		44%	None			0%
Slight		34%	Slight			18%
Moderate		10%	Moderate			25%
Very/extremely strong		22%	Very/extremely strong			57%
Hard to keep from using EC			Hard to keep from smoking			
Yes		36%	Yes			58%
No		64%	No			42%
*WHEN HAVE NOT USED [EC; tobacco] for a while OR when you tried to stop [vaping; smoking] …*						
Irritable because can’t use EC			Irritable because can’t smoke			
Yes		32%	Yes			73%
No		68%	No			27%
Nervous because can’t vape			Nervous because can’t smoke			
Yes		32%	Yes			66%
No		68%	No			34%
Composite Dependence Scores						*p-*value*
Heaviness of Vaping Index [32]	Mean (SD)	2.07 (1.76)	Heaviness of Smoking Index [33]	Mean (SD)	2.36 (1.66)	.2155
PSDI-EC [32]	Mean (SD)	6.42 (4.84)	PSDI-smoking [32]	Mean (SD)	11.02 (5.00)	<.0001

*EC* Electronic Cigarette

*PSDI* Penn State Dependence Index

Missing = 10 for Heaviness of Vaping Index, 12 for PSDI-EC, 2 for Heaviness of Smoking Index, and 3 for PSDI-smoking

*Paired t-test on the differences between the vaping dependence scores and smoking dependence scores

**Fig. 1:**
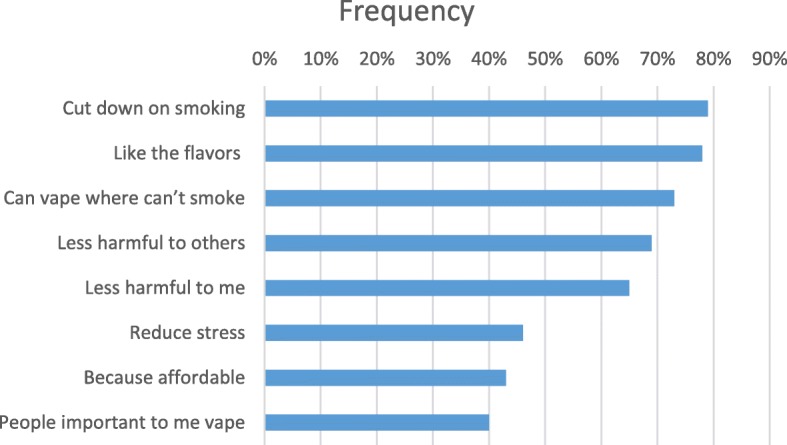
Reasons for vaping among American Indian dual EC and cigarette users

Reasons for vaping are shown in Fig. 1. The top three most commonly endorsed reasons for vaping were to cut down on smoking, liking EC flavors, and being able to vape in places wherein smoking is not allowed. Perceiving less harm to others or to oneself compared with smoking were the next most frequent responses. Less than one-half endorsed using EC to reduce stress, reduce cost, or because other persons important to them used EC. Other reasons (write in) for EC use were endorsed by only two (4.5%) participants.

## Discussion

In this cohort of adult AI persons who smoke, the prevalence of dual use, defined as using EC on some or all of the past 30 days, was 12%. This definition of dual use is similar to other longitudinal studies [7, 36] and helps to exclude recent experimenters or infrequent users [31]. Other studies vary widely in estimates of prevalence of dual use by smokers. In a 2014 study, nearly 52% of a cohort of smokers used ECs either daily or more than just a few days [7]. However, in a 2013 study, prevalence of EC use more than 50 times during lifetime was only 3.8% among smokers [37] and among current smokers in the Current Population Survey in 2014, regular EC use among smokers was 3.6% [38]. Definitions of dual use may vary enough to limit our ability to directly compare prevalence estimates between different cohorts, and the rapid rise in EC use also limits comparison with older studies. Standardized definitions of dual use will be useful in future studies.

### Vaping patterns

Vaping 10 or more times per day was reported by one-third of the dual users. Very few studies report on the frequency of vaping by dual users, and often use different measures [39]. Given the continued debate regarding utility of EC in smoking cessation [40] and evidence of no clear benefit of dual EC and cigarette use [17], frequency of vaping needs more exploration by this group of smokers. Again, standardization of measures of vaping frequency will help in comparing future studies.

First generation (cigalike) ECs were the least often used products and the second-generation “tank” products the most often used. Second- and third-generation products allow users to customize their product, unlike the first generation cigalikes [41], in ways that likely improve the nicotine delivery [22, 42].

### Nicotine content of vaping products among dual users

While the majority of participants used ECs that contained nicotine, nearly 20% either did not or were unsure if they did. In another study, of 399 adult EC users who were current smokers in 2015, 337 (80.7%) used EC containing nicotine, with 19% not [43]. While many smokers who also use ECs do so to reduce smoking, several EC users did not know the nicotine content of their EC product, or even if the product contained nicotine. If ECs are to replace, rather than supplement combustible cigarettes, nicotine content may be an important factor.

#### Measures of EC dependence

Measures of EC dependence were relatively low compared to measures of cigarette dependence among these dual users, similar to findings in other studies [32, 39, 44, 45]. In our study, all participants were current smokers at baseline, so EC dependence among these dual users are not directly comparable to studies of exclusive EC uses. In addition, a relatively higher proportion of dual EC users had missing data for the vaping dependence scales, but the effect of this potential bias is unclear. Smoking dependence questions preceded vaping dependence questions in our survey and the similarity in questions and format may have confused participants. Whether ECs had reduced these respondents’ dependence on cigarettes could not be assessed in this study.

#### Reasons for vaping

Flavoring was the most often endorsed reason for vaping. The impact of flavoring has never been reported for AI who smoke and use ECs. The role of flavoring in use of EC products is of increasing interest. A recent study found that flavors influenced nicotine exposure through flavor liking, but also contributed to heart rate acceleration, and nicotine titration [46]. In the US, sales of flavored EC products have greatly increased, including in Oklahoma [47]. In one study, adult smokers’ interest in flavored EC was modest, but among the smokers who also use ECs, EC use was most affected by flavor [48]. In contrast, flavoring was only infrequently (14.7%) cited as a reason for EC use by current smokers in a national survey [5].

Other than using EC to stop smoking, the convenience of vaping in places where smoking was not allowed was another leading reason for vaping. More than half cited their perception of EC being less harmful to others, and stress, affordability, or others’ vaping were among the least common. Reasons for dual use of EC among smokers usually include desire to quit smoking [49, 50], and perceptions of less harm than cigarettes [49, 51], but other factors are less frequently assessed.

#### Limitations

A small, convenience and clinic-based sample limits our study. In addition, patterns of tobacco use vary across AI communities in the US, and findings from this study may not reflect use in other regions. Nonetheless, this pilot study took place in a region with high prevalence of tobacco use and provides a unique snapshot of AI dual users and their vaping habits. Larger, population based studies will greatly help to elucidate regional differences in the effects of ECs upon the smoking habits of AI people. As EC technology continues to evolve rapidly, the types of EC products used in 2016 may not reflect currently used EC products. Follow up studies are needed to assess changes in use over time.

## Data Availability

The data that support the findings are available from Cherokee Nation, but restrictions apply to the availability of these data. These data were used under agreement for the current study, and are not publicly available. Data are, however, available from the authors but only with explicit permission of Cherokee Nation.

## Appendix

The Penn State Electronic Cigarette Dependence Index [32] as adapted for this study.

1. How many times per day do you usually use your electronic cigarette? (Scoring: 0–4 times/day = 0, 5–9 = 1, 10–14 = 2, 15–19 = 3, 20–29 = 4, 30+ = 5),
2. How soon after you wake up do you first use your e-cig or vape? (Scoring: 0–5 min = 5, 6–15 = 4, 16–30 = 3, 31–60 = 2, 61–120 = 1, 121+ = 0),
3. Do you sometimes awaken at night to vape or use your e-cig? (Yes = 1, No = 0),
4. How many nights per week do you typically wake up to vape or use your e-cig? I never wake up at night to vape, less than one night per week, 1 night per week, 2 nights per week, 3 nights per week, 4 or more nights per week. (Scoring 0–1 nights = 0, 2–3 nights = 1, 4+ nights = 2)
5. Do you vape or use an e-cig now because it is really hard to quit? (Yes = 1, No = 0)
6. Do you ever have strong cravings to use an e-cig or vape? (Yes = 1, No = 0)
7. Over the past week, how strong have the urges to vape or use an e-cig been? None, slight, moderate, strong, very strong, extremely strong. (Scoring: None/slight = 0, Moderate/Strong = 1, Very Strong/Extremely strong = 2)
8. Is it hard to keep from using an e-cig or vaping in places where you are not supposed to, such as restaurants, hospital/clinics, or other public areas? (Yes = 1, No = 0)
9. When you haven’t used an e-cig or vaped for a while or when you tried to stop … Did you feel more irritable because you couldn’t vape or use an e-cig? (Yes = 1, No = 0)
10. When you haven’t used an e-cig or vaped for a while or when you tried to stop … Did you feel nervous, restless, or anxious because you couldn’t vape or use an e-cig? (Yes = 1, No = 0)

## Abbreviations

AI: American Indian
AN: Alaska Native
CDIB: Certificate of Degree of American Indian or Alaska Native Blood
EC: electronic cigarette
US: United States
